# MEG Correlates of Learning Novel Objects Properties in Children

**DOI:** 10.1371/journal.pone.0069696

**Published:** 2013-07-31

**Authors:** Charline Urbain, Mathieu Bourguignon, Marc Op de Beeck, Rémy Schmitz, Sophie Galer, Vincent Wens, Brice Marty, Xavier De Tiège, Patrick Van Bogaert, Philippe Peigneux

**Affiliations:** 1 UR2NF - Neuropsychology and Functional Neuroimaging Research Group at CRCN - Center for Research in Cognition and Neurosciences, and UNI - ULB Neurosciences Institute, Université Libre de Bruxelles (ULB), Brussels, Belgium; 2 LCFC - Laboratoire de Cartographie Fonctionnelle du Cerveau, Hôpital Erasme, Brussels, Belgium; Baycrest Hospital, Canada

## Abstract

Learning the functional properties of objects is a core mechanism in the development of conceptual, cognitive and linguistic knowledge in children. The cerebral processes underlying these learning mechanisms remain unclear in adults and unexplored in children. Here, we investigated the neurophysiological patterns underpinning the learning of functions for novel objects in 10-year-old healthy children. Event-related fields (ERFs) were recorded using magnetoencephalography (MEG) during a picture-definition task. Two MEG sessions were administered, separated by a behavioral verbal learning session during which children learned short definitions about the “magical” function of 50 unknown non-objects. Additionally, 50 familiar real objects and 50 other unknown non-objects for which no functions were taught were presented at both MEG sessions. Children learned at least 75% of the 50 proposed definitions in less than one hour, illustrating children's powerful ability to rapidly map new functional meanings to novel objects. Pre- and post-learning ERFs differences were analyzed first in sensor then in source space. Results in sensor space disclosed a learning-dependent modulation of ERFs for newly learned non-objects, developing 500–800 msec after stimulus onset. Analyses in the source space windowed over this late temporal component of interest disclosed underlying activity in right parietal, bilateral orbito-frontal and right temporal regions. Altogether, our results suggest that learning-related evolution in late ERF components over those regions may support the challenging task of rapidly creating new semantic representations supporting the processing of the meaning and functions of novel objects in children.

## Introduction

During their development, children have to rapidly assimilate the meaning of countless novel objects. Functional properties are powerful determinants in the way novel objects are categorized and learned ([1]; for a review see [2]). For instance, it has been shown that 14-month old infants are more likely to group objects into a category when functional commonalities between objects have been demonstrated during learning than when objects merely present perceptual similarities [3]. Furthermore, when functions of novel objects are demonstrated, children as young as 2 years old can generalize the name of these objects to novel objects presenting similar functions [4]. Hence, learning about meaning and functions of novel objects crucially contributes to the development of semantic representations, eventually providing a broad cognitive and conceptual basis onto which children can map words of language [5], [6]. In this respect, determining the functional neuroanatomical patterns supporting the retrieval of newly learned semantic properties about objects and their functions in healthy children might provide insights to understand the pathophysiology of conditions leading to abnormal language development (e.g. specific language impairment). To the best of our knowledge however, few existing neuroimaging studies have focused on the *learning* of new functions of objects. Rather, the functional properties associated to novel objects have been studied in adults either in terms of action/manipulation (i.e. “how objects are used”) [7] or in the context of the acquisition of novel lexical representations in the vocabulary [8]–[11], rather than in terms of abstract functionalities about objects (i.e. “for what purpose objects are used”).

It is assumed that functional properties of familiar objects belong to semantic memory [12], the component of declarative memory where knowledge-based information and concepts are stored independently of their spatio-temporal context of acquisition [13]. Furthermore, it has been suggested that abstract functional features of semantic representations may be merely grounded in multimodal integrative brain regions (for a review see [14]). Accordingly, Yee et al. [15] showed using functional magnetic resonance imaging (fMRI) that the degree of similarity between the functional features of two objects in a word pair ("functionality" being defined here as the purpose for which an object is used; e.g. a flashlight and a lantern have the same function) was specifically correlated with activity in the left superior frontal, medial temporal and middle temporal regions during a reading task. The latter temporal regions were characterized as multi-modal areas integrating information from unimodal cortices (for a review see [16]), suggesting that the temporal lobe may support the encoding of functional properties by integrating information about shape and manipulation incoming from other areas. Additionally, Ishibashi et al. [17] showed using repetitive transcranial magnetic stimulation (rTMS) that virtual lesions over the left anterior temporal lobe eventually slow down similarity judgments between tool functions during a task in which participants decide whether two words share the same functional or manipulation features, hence mirroring performance observed in mild semantic dementia patients [18]. Conversely, rTMS over the left inferior parietal lobule affect manipulation judgments about objects [17], mirroring apraxic patients with parietal lobe damage [19].

**Figure 1:**
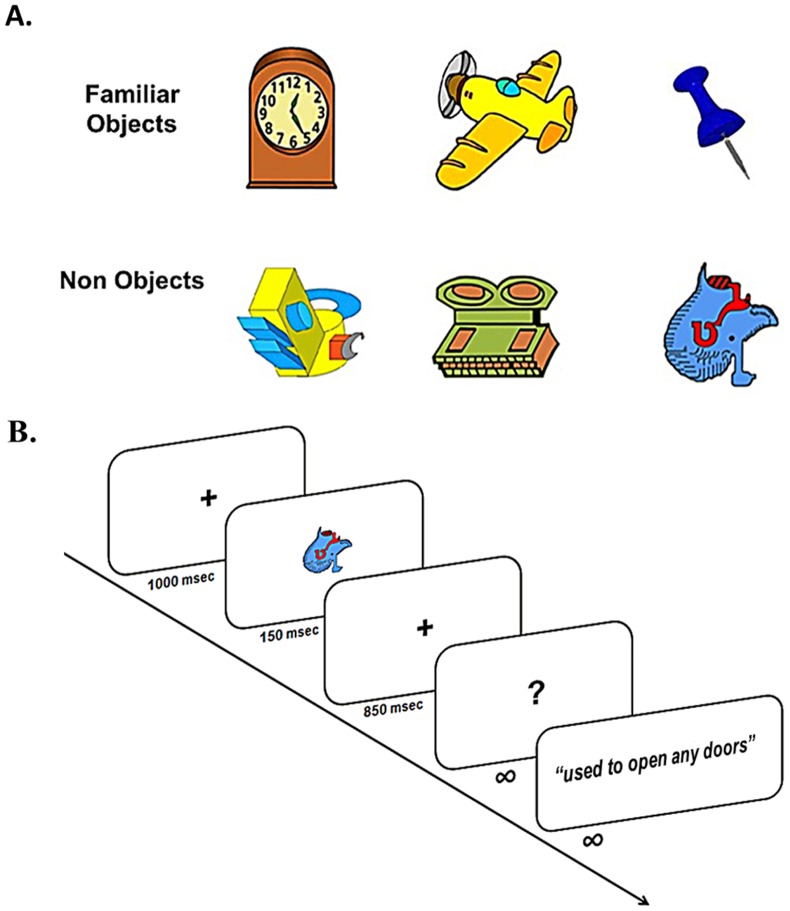
Experimental protocol. (a) Experimental stimuli. Sample illustrations of the 50 familiar objects (FO) and 100 novel non-objects (LNO, UNO) used in this study. Functional properties were taught for half of the 100 non-objects during a behavioral learning session after MEG session 1 (S1), thus becoming learned non object (LNO) at session 2 (S2). No associations were taught for the other 50 non-objects that remained unknown (unlearned) non-objects (UNO) at both sessions 1 and 2. (b) MEG picture naming/defining task: children were asked to provide the name and/or a definition of the object presented on the screen after the appearance of the question mark 1 sec after stimulus presentation, or to signal if the object was unknown.

In this perspective, the processing of well stored functional features might be represented over the temporal lobe, allowing flexible and integrative mechanisms to deal with abstracts concepts that are not linked to a particular modality in adults ([15], [17], for a review see [14]) but further research is needed to understand the cerebral processes subtending the learning of new semantic representations. The present magneto-encephalography (MEG) study is aimed at describing the neurophysiological processes subtending the acquisition of functionalities and meanings for novel objects in children. To do so, we recorded evoked magnetic responses elicited by the visual processing of novel non-objects (to-be-learned non-object; LNO) before (Session 1; S1) and after (Session 2; S2) 10-years old healthy children learned their functional properties through short verbal definitions during a behavioural session. Additionally, 50 familiar real objects (FO) and 50 other unknown non-objects (UNO) for which no functions were taught were presented at both MEG sessions (Fig. 1).

## Materials and Methods

### Participants

Sixteen healthy children and their parents gave written informed consent to participate in this experiment approved by the Biomedical Ethics Committee of the Erasme Hospital-Université Libre de Bruxelles. All children were native French speakers with no known learning, language or neurological problem. Five children had to be excluded due to poor MEG signal quality or EMG/EOG artefacts (see *MEG Data Analysis* below). Consequently, the final sample was composed of eleven healthy children (3 boys; mean age: 10.21 years; range, 8.5–12.4 years; all right handed; laterality score mean ± s.d. = 82.72±14.89; [20]).

### Pictures and Verbal Stimulus Material

One hundred fifty stimuli were designed by the same artist and paired using Photoshop™ 2 in terms of contrast, brightness, size and colours. Stimuli included single 2D coloured outline drawings of 50 familiar objects (FO) and of 100 unfamiliar non-objects (see example Fig. 1a), the latter set randomly divided between 50 to-be-learned non-objects (LNO) and 50 to-remain-unknown non-objects (UNO). Non-objects were adapted from two databases of non-objects [21], [22] and matched to the physical properties of familiar objects (FO). FO were selected on the basis of their high incidence in the receptive and expressive vocabularies of French-speaking young children (high lexical frequency, Lexique3 [23]). FO included both artefacts (e.g., airplane, thumbtack) and natural (e.g. cherry, palm) stimuli, equally distributed, had intermediate emotionality level and were highly concrete and imaginable.

All objects were validated in a behavioural pilot study conducted in a separate group of children (N = 37), who were asked to perform the picture naming/defining task used in our MEG experiment (see details about the procedure below). This pre-test revealed, on the one hand, that most children usually knew both the function and the name of each FO object and, on the other hand, that none of the 100 non-objects was associated with any specific meaning (or that a meaning might be erroneously deduced from the shape of the non-object). Each of the 100 non-objects was then randomly associated with a definition explaining the « magic » function/properties of the object. The definitions, in French, were 4 to 7 words long (e.g., with this object you can “see through the walls”, “open any doors”, “stop the rain”, “read people's thoughts”, “quickly heal wounds” …).

Four lists of 50 LNO stimuli (randomly selected from the set of 100 non-objects) were created and assigned to each child for the learning phase in counterbalanced order.

### Experimental Task and Procedure

During each MEG session (S1 and S2), all stimuli were presented twice to enhance signal-to-noise ratio, in a randomized order. During MEG recording, stimuli appeared on a projection screen located 1 m in front of the child, fitting in a 3°×3° area in the central visual field. Each picture was shown for 150 msec, followed by a 850 msec blank screen then by a question mark prompting the child to say aloud its response (name and/or definition of the object, or “skip” if unknown), which remained on screen until a response was provided. The next trial was initiated 1000 msec after the subject's response (Fig. 1b). A delayed naming/defining response procedure (as opposed to an immediate verbal response that would have allowed to measure reaction times) was chosen to equalize as much as possible the process of word retrieval between conditions prior to actual vocalization, and to reduce mouth movement artefacts in MEG recording during the post-stimulus period of interest.

In the pre-learning session 1 (S1), evoked MEG responses were recorded during visual presentation of the 50 familiar (FO) real objects and of the 100 novel non-objects (50 LNO and 50 UNO). Immediately afterward, children learned outside of the scanner the “magical” functional properties of a subset of 50 unfamiliar non-objects [i.e. the to-be-learned non-object; LNO] until reaching a 75% learning criteria. In order to mimic as close as possible an ecological situation of learning novel objects during childhood, a non-word label was also provided for each to-be-learned non-object (e.g. “*This object is a “kuvl
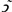
”, you can use it to see through the walls”*). However children were not mandatorily required to memorize the non-word labels which were not included in the analyses. Label names for the 50 to-be-learned non-objects (LNO) were created using the non-words generator “Lexique toolbox” (http://www.lexique.org/toolbox/SiteLexiqueenvoie/; [23]). Non-word labels were paired to the names of FO stimuli in terms of number of syllables, phonotactic regularities and bigrams position. Post-learning session 2 (S2) was then conducted immediately after the end of the behavioural learning session. Evoked MEG responses were again recorded using the same procedure than in S1 using the previously shown 50 familiar objects [FO] and 50 unknown (and unlearned) non-objects [UNO], and the 50 novel non-objects now associated with novel functional properties during the learning session [LNO]. A detailed presentation of Material and Methods is provided as Supplementary Information (SI) in Supporting Information S1.

### MEG Data Acquisition

MEG recording took place in a light-weight magnetically shielded room (MaxShield, Elekta Oy, Finland) using a helmet-shaped 306-channel whole head neuromagnetometer (Vectorview, Elekta Oy, Finland), the characteristics of which are described elsewhere [24]. The system contains 102 triple sensor elements, each composed of two orthogonal planar gradiometers and one magnetometer. Planar gradiometers best detect signal directly above an active cortical area, whereas magnetometers are best suited to detect deeper sources activity. Band-pass was 0.1–300 Hz and sampling rate set at 1 kHz. Eye movements and blinking artefacts were monitored with electrodes attached vertically around the eyes. Mouth movements were monitored using two electrodes vertically around the mouth (orbicularis oris muscle). Head position inside the MEG helmet was measured before each recording session then continuously monitored using four head tracking coils (two at the front and two behind the ears [25]). The locations of the coils with respect to anatomical fiducials (nasion and two tragus) were determined using an electromagnetic tracker system (Fastrak, Polhemus, Colchester, VT, USA).

### MEG Data Analyses

Analysis of MEG recordings aimed at revealing changes in neurophysiological responses to novel non-objects, from before to after learning their functional properties while controlling for stimulus repetition effects. Only items having elicited a correct response were used in the analysis of MEG data (mean percentage of incorrect responses was below 25%, thus preventing error-related analyses).

External noise was removed from the MEG data using the temporal extension of the Signal-Space Separation (SSS; [25]) implemented within the MaxFilter software (Elekta Oy, Finland). In the SSS method, the field patterns originating from outside a sphere encompassing the sensor array are separated from those originating inside, which contains the head and the brain activity of interest [26]. As compared to adults, the smaller head size of children allows for larger head movements inside the measurement helmet during the experiment, which can eventually lead to loss of spatial details and inaccurate localization of the brain activity [27]. Therefore, we used the SSS method that extrapolates the measured signals (each 200 msec) from different head positions of the child to a virtual array fixed with respect to its head [25] in order to compensate the MEG signal for head movements (for further details see [27]). The SSS method was also used to align all subject’s heads between session and between subjects in the same sensor space (i.e. referred to the initial head’s position coordinates of one optimally placed subject) in order to provide a suitable context for between-subjects statistical analyses.

Subsequent pre-processing and analysis steps were performed using SPM8 (version 8.6 (SPM8) 27-Feb-2012, Wellcome Trust Centre of Neuroimaging, London: http://www.fil.ion.ucl.ac.uk/spm/) implemented in MATLAB 9 (Mathworks, Sherbom, MA). MEG signals (from gradiometers and magnetometers) were filtered (fifth-order Butterworth band-pass filter at 0.5–40 Hz) and baseline-corrected epochs were extracted from 200 msec prior to the onset of each picture (baseline −200 to 0 msec) to 1100 msec post-stimulus onset. Data were then downsampled at 250 Hz. Epochs contaminated by eye (EOG) or mouth (EMG) movements superior to, respectively, 150 µV and 300 µV were rejected. Epochs during which a MEG sensor signal exceeded the level of 2000 fT/cm for planar gradiometers and 4000 fT for magnetometers were also rejected. Sensor data were then averaged per subject and condition (FO, LNO and UNO at S1 and S2 separately). Resulting averaged event-related fields (ERFs) files were then converted into 3D spatiotemporal volumes by stacking 2D linearly interpolated images into peristimulus time, and spatially smoothed (9 * 9 mm * 20 msec FWHM Gaussian kernel). Single image per condition for gradiometers (generated from root-mean-square values combining the two planar gradiometers at each location) were then entered in a factorial design model using conventional SPM procedures [28] at the random effect level with within-subject Session (pre- [S1] vs. post- [S2] learning) and Object type (familiar objects [FO], to-be-learned/learned non-objects [LNO] and unknown non-objects [UNO]) factors. An explicit temporal mask was included to restrict statistical analysis within a specific time window of interest 50–950 msec post-stimulus onset.

Planned comparisons (*t-*contrasts) of interest tested for between-sessions differences in ERFs for non-objects first unknown (S1) then learned (S2) at each voxel in the sensor space and time (LNO [S1 vs. S2]). To control for the possible confounding effect of stimulus repetition between sessions, an exclusive mask (p^uncorrected^ <.05) was computed on between-sessions differences for untaught non-objects (UNO S1 vs. S2). Hence, the exclusive mask removed from our analysis of interest (LNO S1<S2) all voxels in which activity significantly differed between S1 and S2 (potential repetition effect) for unknown non-objects that were unchanged between sessions (i.e. never learned; UNO S1 vs. S2). Resulting statistical parametric maps revealed suprathreshold spatiotemporal clusters characterized by 2D sensor-space location coordinates (x, y mm) and peak time (in milliseconds post-stimulus onset). Statistical significance was set at p≤.05 at the cluster level, corrected for multiple comparisons in the whole sensor-space volume using Gaussian random field theory family-wise error (FWE) as conventional using SPM [29].

Anatomical sources were reconstructed using the imaging (or distributed) method implemented in SPM8. This approach results in a spatial projection of sensor data into (3D) brain space and considers brain activity as comprising a very large number of dipolar sources spread over the cortical sheet, with fixed locations and orientations, rendering the observation model linear, the unknown variables being the source amplitudes or power [29]. Evoked activity for each condition at each session per individual was estimated for each dipolar source separately for the peristimulus time windows of interest. Source reconstruction proceeded first by coregistering a 8196 vertex cortical mesh template (as provided in SPM8 and defined in the MNI stereotaxic space) to the sensor positions using three fiducial marker locations identified on each individual child's MRI [30]. We then used a single shell head model for the forward computation of the gain matrix of the lead field model [31]. Source estimates were obtained on the cortical mesh via inversion of the forward model [32], [33], by modeling the covariance components using the multiple sparse prior method on both gradiometers and magnetometers [34], in conjunction with a greedy search [35] as implemented in SPM8. Results were then temporally windowed based on the timing of significant effects in the sensor space (as suggested in previous MEG studies using SPM8, (e.g. [36], [37]); i.e. 530–690 and 700–760 msec, corresponding to suprathreshold time windows (±20 msec) found over left frontal and right temporal sensors respectively; Table 1, see Results), producing a series of individual inversions (i.e. separate source reconstructions) for each participant.

**Table 1:** Modulations of brain responses (ERFs) in the sensor space, associated with the learning of novel objects properties (LNO S1<S2).

(x, y, msec)	Sensor space region	*Z*	Peak-level (FEW-corr)	^K^E *cluster*	Cluster-level (FEW-corr)
−8, 56, 656	Left frontal sensors	4.02	0.216	579	0.016
4, 72, 724	Right frontal sensors	3.67	0.534	449	0.038
64, −30, 740	Right temporal posterior sensors	4.11	0.16	403	0.051
64, 2, 572	Right temporal anterior sensors	3.92	0.289	452	0.037

Learning-related modulation of cerebral activity elicited by defining novel objects properties during the MEG task (LNO S1<S2) at the population level in the sensor space. x, y, and msec are 2D sensor-space locations (x, y) and peak time in milliseconds (msec). Z = Z-statistic value. KE cluster = cluster extent of the activation (in number of voxels). Reported activations are statistically significant at the cluster-level (p^corr^ ≤0.05 after corrections for multiple comparisons in the whole brain volume).

Resulting individual contrast images (all object conditions at S1 and S2) in NIFTI format were spatially smoothed using a Gaussian kernel of 12 mm FWHM, then entered in a second-level corresponding to a random-effects analysis [38]. Analyses and contrasts using the general linear model were the same than in the sensor space, to the exception that source images were defined within a specific time window (see above): individual NIFTI images were entered in a factorial design modeled at the random effect level with within-subject Session (pre- [S1] vs. post- [S2] learning) and Object type (familiar objects [FO], to-be-learned/learned non-objects [LNO] and unknown non-objects [UNO]) factors. Planned comparisons (*t-*contrast) of interest tested between-sessions differences for to-be-learned non-objects (LNO [S1 vs. S2]), masked exclusively for repetition effects on UNO stimuli (p^uncorrected^ <.05; UNO S1 vs. S2). The resulting set of voxel values constituted a map of t statistics [SPM(T)], reported significant at p^uncorrected^ <.005 or p^svc^ <.05 after correction in a small volume of interest (radius 10 mm) drawn around coordinates of interest taken from the literature. F-contrasts on LNO objects illustrating the main effects of pre- and post- learning (S1 and S2), that constitute the statistical contrast of interest (LNO S1<S2) were computed for illustrative purpose. The resulting set of voxel values constituted a map of F statistics [SPM(F)], significant at p≤.05 corrected for multiple comparisons in the whole brain volume using whole-volume Gaussian random field theory.

## Results

### Behavioural Results

During the behavioural learning session occurring between MEG sessions S1 and S2, children learned the short definitions describing the “magical” functions of the 50 to-be-learned LNO stimuli, randomly selected within the set of 100 unfamiliar non-objects presented at S1. One to three presentations of the LNO stimuli were needed before reaching the 75% learning criterion (mean percentage of correct responses ± standard deviation at the end of learning: 85.27% ±6.76%). The duration of the learning session never exceeded 1 hour.

During each MEG session (S1, S2), the set of 150 stimuli was presented twice in random order. Responses were considered correct when either the name or an appropriate definition was provided for FO stimuli at S1 and S2 (accuracy score at S1 = 94.27% ±5.58, at S2 = 95.36% ±4.63) and only when learned definition was provided for LNO stimuli at S2 (76.1% ±10.5), whereas unknown non-objects were considered as correct when they were identified as such (i.e. “*I skip*”; LNO at S1 = 90.72% ±12.53; UNO at S1 = 88.1% ±14.7 and S2 = 87.7% ±14.64). Accuracy scores were similar between S1 and S2 both for FO and UNO stimuli (Student paired t-tests, *ps* >.07), and higher at S2 for FO than for LNO stimuli (*t* (10) = 4.5; *p*<.0005). Children mostly responded by providing names for FO stimuli (98% naming answers) whereas definitions of magic properties but not names were provided for LNO stimuli (100% function answers).

### Learning-dependent Neurophysiological Changes in Novel Object Processing

Our main analysis aimed at highlighting learning-dependent changes in the processing of LNO stimuli (i.e. before vs. after learning their functions) during the period preceding the occurrence of the delayed verbal response (i.e. from stimulus onset to 950 msec post-stimulus onset, see Fig. 1), controlled for visual repetition effects due to the presentation of the same LNO objects at S1 and S2.

**Figure 2:**
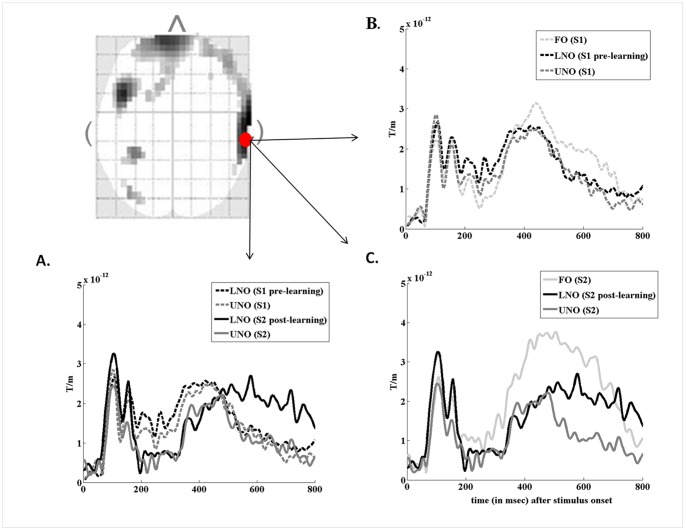
Learning-related changes in evoked-related fields (ERFs) over the right temporal region (analysis in sensor space). Two dimensional statistical map (left, top side) showing sensor-space differences (t-test, p<.001) in field intensities of LNO evoked-responses between sessions (LNO S1< S2), at 740 msec post-stimulus onset masked by the repetition effect for UNO objects (S1 vs. S2). Red points indicate the location of MEG gradiometers (26 33 and 26 32) eliciting evoked responses represented on Figure 2 (a, b and c). (a) Grand average time courses of ERFs for LNO and UNO non-objects at S1 (LNO: black hyphenated line, UNO: gray hyphenated line) and S2 (LNO: black line; UNO: gray line). Significant differences are identified 720–740 msec post-stimulus onset. (b) Grand average time course of ERFs for FO (light gray hyphenated line), pre-learning LNO (black hyphenated line) and UNO (gray hyphenated line) at S1. (c) Grand average time course of ERFs for FO (light gray line), pre-learning LNO (black line) and UNO (gray line) at S2.

Analysis in the sensor space disclosed a learning-dependent modulation of ERFs elicited by LNO stimuli (LNO S1< S2, masked exclusively by between-sessions differences for unknown non-objects UNO S2 vs. S1; see Fig. 2a and Table 1), developing 500 to 800 milliseconds post-stimulus onset over bilateral frontal (−8 56 mm in standard coordinate space and 656 msec peak post-stimulus onset; 4 72 mm and 724 msec) and right temporal regions (64 −30 mm and 740 msec; 64 2 mm and 572 msec; all ps ≤.05 at the cluster level corrected for multiple comparisons; clusters extent >403; Z-values >3.67). For the sake of completeness we additionally computed a “Session” (S1, S2) by “Condition” (LNO, UNO) interaction. Resulting statistical parametric maps disclosed effects in similar locations with a suprathreshold spatiotemporal cluster over the right temporal sensors (64, −30 mm, 740 msec; p<.001, Z = 3.11). Additionally, small volume corrections (SVC^10 mm^) computed around the three other sensor space coordinates reported in Table 1 (−8, 56 mm, 656 msec; 4, 72 mm, 724 msec; 64, 2 mm, 572 msec) also disclose effects on the same gradiometers (respectively, −8, 50 mm, 652 msec, Z = 2.32,p^corr^ = .045; 4, 72 mm, 720 msec; Z = 2.81, p^corr^ = .021; 68, −3 mm, 568 msec; Z = 2.64, p^corr^ = .021).

Figure 2a illustrates the grand average (over the population) peristimulus time course of evoked magnetic responses at the right posterior temporal sensor located nearest to the peak statistical coordinate (64 −30 mm), for non-objects before and after children learned their functional properties (LNO), and for other non-objects (UNO) that remained unknown at both sessions. Data inspection confirms the development of a late component of increased amplitude 500–800 msec post-stimulus onset after learning the functions of non-objects (LNO S1<S2; significant between-sessions differences 720–740 msec post-sti22mulus onset). Conversely, the time course for unknown non-objects remained closely similar between sessions (UNO S1 vs. S2), discarding the hypothesis that observed changes for LNO in the right posterior temporal sensor are merely due to a stimulus visual repetition effect. Looking at other suprathreshold coordinates (Table 1), we confirmed similarly noticeable changes in the amplitude of late components after learning objects functions over left frontal sensors (−8 56 mm; 520 to 720 msec post-stimulus onset; suprathreshold differences 552–656 msec; see Fig. S1 in Supporting Information S1). However, data inspection failed to disclose clear-cut changes in the time course and amplitude of evoked magnetic responses over the right frontal (4 72 mm) and right temporal anterior (64 2 mm) sensors, that were less markedly different from evoked responses to never taught non-objects (UNO) (Table 1; Fig. S2 in Supporting Information S1).

Additionally, figures 2b and 2c illustrate grand averaged evoked responses (over the right posterior temporal sensor; 64 −30 mm spatial coordinates) for familiar objects [FO], learned [LNO] and unfamiliar [UNO] non-objects separately at pre- (S1) and post- (S2) learning sessions. Data inspection further indicates that FO stimuli elicited a late component of higher amplitude (400–750 msec post-stimulus onset at S1; 300–700 msec at S2) than both LNO and UNO at pre-learning S1, despite earlier (<400 msec) similar responses for all three objects type. At post-learning S2 however, freshly learned non-objects (LNO) elicited an intermediate increase in late components, closer to FO although still of lower amplitude (400–700 msec post-stimulus onset), but also of higher amplitude than for UNO objects (range 500–800 msec). Comparable time courses were observed over the left frontal sensor suggesting that at least in these two spatial locations in sensor space, late evoked magnetic responses might be associated with the development of new semantic functional knowledge about novel objects. Although we describe ERFs for familiar objects, specific statistical comparisons with learned non-objects were not performed here given the aforementioned qualitative differences in response between these categories (i.e. reporting names vs. functionalities, respectively).

**Figure 3:**
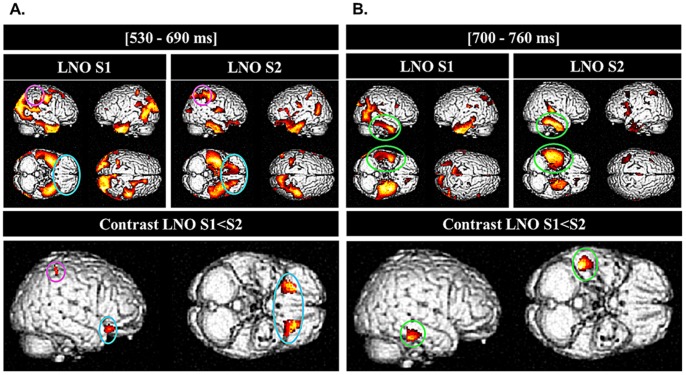
Learning the Functions of Novel Objects: Brain Sources. Top panels: Source reconstruction of neural activity elicited during picture definition in each session separately for LNO objects during significant time windows identified in the sensor space (a) 530–690 ms, (b) 700–760 ms (left subpanel, LNO S1 pre-learning session; right subpanel, LNO S2 post learning session; displayed at p<.001 uncorrected). Colors code the value of the F statistic associated with each voxel. Bottom panels: source reconstructions for neural activity underlying learning-related effects (LNO S1<S2: P<.005 uncorrected) during the same time windows: (a) bilateral activations over the orbito-frontal gyri and the right parietal posterior gyrus at 530–690 msec; (b) right lateral occipito-temporal and infero-temporal activations at 700–760 msec. Colors code the value of the t statistic associated with each voxel.

Comparisons computed on reconstructed sources for learned non-objects (LNO) over the peristimulus time window 530–690 msec (based on a priori time windows derived from suprathreshold time windows (±20 msec) of the statistical findings in sensor space, see above) disclosed learning-related increased source amplitude at S2 (vs. S1) bilaterally in the orbito-frontal cortex (peak voxels in standard stereotactic MNI brain coordinates −18 30 −6 mm; 22 30 −6 mm) and in the right posterior parietal cortex (42 −42 58 mm; all *p*<.005 uncorrected; Table 2; Fig. 3a). Fig. 3a also displays reconstructed sources for LNO objects at each session separately, indicating that activations over the bilateral orbito-frontal (in blue) and right lateral parietal (in pink) areas were not present at the pre-learning session S1 (even at largely permissive statistical threshold p^uncorr^<.01). Also, activity in bilateral occipito-temporal regions (including bilateral fusiform gyri) dissipated at S2 (LNO objects S1> S2; peak voxels: −40 −68 −24 mm; 48, −56, −6 mm; *Z* >2.78; p^uncorr^ ≤.003; Table S1 in Supporting Information S1). Finally, a conjunction analysis [39] revealed common LNO-related activation patterns at S1 and S2 in bilateral temporal gyri (−40 −2 −30 mm; 42 −4 −30 mm; Z >4.35; all *p^corr^*<.05) and posterior cingulate cortex (−22 −30 38 mm; 26 −22 36 mm; Z >4.45; Table S2 in Supporting Information S1).

**Table 2:** Modulation of brain activity in the source space, associated with the learning of novel objects properties (LNO S1<S2), masked exclusively for between-sessions repetition effects for untaught non objects (exclusive mask p^uncorr^ <.05; UNO S1 vs. S2).

Time window (ms)	*Voxel-level*						*Cluster extent*	
	Region	Side	x	*y*	*z*	*Z*	^K^E *cluster*	*Additional activated regions in the cluster*
530–690	Middle orbito-frontalcortex (BA 11, 12)	Left	−18	30	−6	2.86	309	–
	Superior orbito-frontalcortex (BA 11, 12)	Right	22	30	−6	2.85	283	–
	Lateral parietal lobule(BA 7)	Right	42	−42	58	2.67	100	–
700–760	Inferior temporal gyrus(BA 20)	Right	46	−34	−10	2.86	341	Right middle temporal gyrus (BA 21)/Right medial temporal area
	Parahippocampal/medialtemporal gyrus* (BA36)	Right	44	−30	−10	2.76	13	

Learning-related modulation of activity elicited by defining novel object’s function during the MEG task (LNO S1<S2) at the population level in the source space. Time windows of sources reconstruction correspond to temporal epochs (±20 msec) during which significant learning-related activations (LNO S1<S2) are statistically identified in the sensor space. *x*, *y*, and *z* are standard MNI coordinates (mm). *Z* = Z-statistic value. ^K^E cluster = cluster extent of the activation (in number of voxels). All reported activations are statistically significant at the voxel-level, *p^corr^* <.005 uncorrected. Activations marked by a * are statistically significant at the voxel-level p^svc^ <.05 after correction in a small volume (radius 10 mm) drawn around coordinates of interest taken from the literature [15], left medial temporal lobe: −36, −27, −15).

Second, comparisons computed on reconstructed sources for LNO objects over the peristimulus time window 700–760 msec (based on a priori time windows derived from suprathreshold time windows (±20 msec) of the statistical findings in sensor space, see above) disclosed a large cluster of learning-related increased source amplitude at S2 (vs. S1) in the right inferior-middle temporal gyri (46 −34 −10 mm; *p*<.005, uncorrected) and in the right medial temporal gyrus (44 −30 −10 mm; p<.05 after correction in a small volume of interest of radius 10 mm) around an *a priori* left medial temporal coordinate associated with the processing of semantic properties about familiar objects functions in a word-reading task conducted in adults using fMRI ([15], see Table 2; Fig. 3). Again, we reconstructed sources for LNO objects at each session separately (Fig. 3b). A conjunction analysis revealed common LNO-related activation patterns at S1 and S2 (in green) in right medio-temporal (42 −26 −8 mm; Z = 4.72; *p^corr^*<.05) and bilateral activation in inferior temporal areas at both sessions (−40 −8 −24 mm; 56 −4 −34 mm; Z >4.40; all *p^corr^*<.05; see Table S2 in Supporting Information S1), however predominant in the left hemisphere during pre-learning S1 (peak voxel: −42 −10 −24 mm, Z = 5.10; p^corr^<.001) then right-lateralized at post-learning S2 (peak voxel: 42 −26 −8 mm, Z = 5.88; p^corr^ <.0001). Indeed, looking at the number of voxels co-activated in each temporal cluster in S1 as compared to S2, the number of voxels activated in the left temporal region compared to right temporal region was reversed between sessions (S1: 1230 (left) vs. 22 (right); S2: 64 (left) vs. 2863 (right); Table S3 in Supporting Information S1). Finally, activity in the right parieto-occipital regions dissipated at S2 (LNO objects S1> S2; 30 −58 26 mm; Z = 3.21; p^uncorr^<.001; Table S1 in Supporting Information S1).

## Discussion

The current study investigated the neurophysiological changes subtending the learning of semantic functional properties of novel, unknown objects, in children. To do so, evoked magnetic responses were recorded during an object definition task administered immediately before and after a behavioural verbal learning session.

At the behavioural level, all children quickly and successfully learned at least 75% of the proposed definitions describing the “magical” functions of 50 non-objects. Although our design was not specifically built to assess this specific ability, this result might tentatively be related to the well known ability that children share with adults [40] to rapidly map new functional meanings to novel objects, even over few expositions. Indeed, if “*fast-mapping*” paradigms have behaviourally demonstrated the capacity of children to quickly learn the meaning of words (as initially demonstrated by Carey and Bartlett [41]), it was subsequently shown that they are equally able to rapidly learn the meaning of objects [42], [43].

At the neurophysiological level, analysis of evoked magnetic responses in sensor space and reconstruction of brain sources disclosed learning-related increases from S1 to S2 in long-lasting late components extending 500–800 msec peristimulus time, first in medial orbitofrontal and right parietal (530–690 msec) then in right medial, middle and inferior temporal (700–760 msec) gyri. Additionally, complementary observations suggest that initially bilateral non-objects related effects (530–690 msec) in the temporal lobes at both sessions turn out at later peristimulus time (700–760 msec) to be left-lateralized before learning functional properties but right-lateralized afterward. This spatiotemporal dynamic of cortical functioning might underlie the transformation of non-objects initially perceived as meaningless during the pre-learning MEG session into objects featuring distinct semantic properties during the post-learning MEG session. In the present study however, it cannot be claimed that learning-related activations exclusively reflect the learning of new functional properties, as other non-functional semantic and episodic elements may have been learned concomitantly. Future research should control for these features, for instance by comparing the obtained effects with those elicited by learning non-functional control information for unknown objects during the intermediate learning session (e.g. “*this object grows on trees*”, “*is found in New Zealand*”, “*is easily breakable*”, …).

Notwithstanding this potential limitation, we surmise here that increased activity for recently learned objects in temporal areas might be specifically involved in the retrieval of newly acquired semantic representations about object’s function, in line with prior findings from adult fMRI and MEG studies. Indeed, it was shown using fMRI that the degree of similarity between the functional features of familiar words specifically correlate with medial temporal lobe activity in a brain location nearby to our own finding, although in the opposite hemisphere [15]. Moreover, a MEG study disclosed similar long-lasting learning-related increased neuromagnetic responses (300–800 msec post-stimulus onset) over the left temporal lobe [9]. These magnetic responses were suggested to be involved in both the retrieval of new lexically-stored phonological and semantic representations of real objects functions. We propose here that the left lateralization of functions-related activations in those prior studies might be due to the fact that functional representations were associated only to real, familiar or newly lexically integrated objects representations. Whereas in the present study, children had to process totally novel semantic representations randomly associated with unfamiliar non-objects, for which functional properties were taught just before recording. Hence, the “virgin” character of the to-be-learned functional semantic representations in the present study (as opposed to real objects) and the short time interval elapsed between learning the novel functions and the MEG retrieval session may have prevented a fully effective integration of these novel representations in the mental lexicon. Rather, they would be still at the state of emerging, unconsolidated representations. We surmise that learning novel semantic representations for unknown, non-objects might have led to the creation of unusual or unfamiliar semantic combinations between wider semantic fields, a process specifically supported by the right hemisphere, in line with studies conducted using divided visual fields [44], event-related potentials [45] and fMRI [46] paradigms. Additionally, it was suggested that the right hemisphere is better suited to cope with novel situations for which no prior representation is available or ready for use, like during the early stage of learning, whereas the left hemisphere would take over at a more advanced stage of learning [47]–[50]. This interpretation would also be congruent with the Coarse Semantic Coding Hypothesis proposing that the right hemisphere is implicated in “coarse semantic coding”, co-activating large semantic fields which may overlap and thus support the generation of distant associations [51], [52]. Although not sufficiently recognized, the role of the right hemisphere in verbal memory and semantic processing has been evidenced in several neuroimaging studies. For example, virtual lesions using rTMS over either the right or the left temporal lobe specifically alter processing time in semantic verbal (words) and visual (pictures) tasks [53], [54]. Also, it was shown using fMRI that preserved verbal memory functions are reorganized within the right medial temporal lobe in adult patients with left temporal medial pathology [55]. Likewise, the incidental learning of new pseudo-words progressively linked to known objects into the mental lexicon is associated both with bilateral middle and superior temporal activations and increased left-right hippocampal connectivity [56].

An alternative explanation would be that increased familiarity with the objects used during the learning session partially contributed to the observed effects, as LNO stimuli were displayed more often than the other objects. In this respect, presenting all stimuli (trained and not-trained) the same numbers of times would have mitigated familiarity as a possible confound. Therefore, it could be argued that the disappearance of source activity observed over the posterior occipito-temporal or occipito-parietal areas in time windows of interest (530–690 msec and 700–760 msec, respectively) may reflect a suppression repetition effect. Indeed, using object repetition paradigms, van Turennout et al. (2000) and Vuilleumier et al. (2002) reported that repeating meaningless (nonsense) and meaningful (real, nameable) objects induces repetition suppression effects in the occipital cortex whereas more anterior regions of the visual object processing stream, like the fusiform gyrus on the ventral surface of the temporal lobes, exhibit repetition suppression effects only to real objects ([57], [58] but see [59] for a review about suppression repetition effects). However, no repetition-related decrease in neural activity was observed for untaught objects (UNO) over bilateral frontal and right temporal regions in the present study, which makes familiarity an unlikely explanation for the reported learning-related effects.

From another perspective, higher levels of difficulty may increase the need for a right hemispheric participation in semantic tasks [60]. According to this view, it is possible that alongside material novelty, higher task difficulty may have contributed to hemispheric laterality differences between our own results and those reported in the Hultén et al. (2009) MEG study [9]. Indeed, Hultén et al (2009) observed similar long-lasting components over the *left* temporal lobe, related to functional learning and additionally predictive of memory performance for novel words up to 10 months later [61]. However, participants were adults and the post-training MEG recording session was administered after several days of intensive training, allowing better and deeper integration of the learned functions in semantic system. Conversely, we scanned here pre-pubertal children after a short 1-hour learning session only. Although they successfully learned the semantic functional properties of novel objects, it is possible that episodic memory processes also participated in the retrieval of those emerging semantic representations. Moreover, further studies are needed to clarify the respective contribution of material novelty and neural maturation processes in the specific recruitment of a learning-related right hemisphere activation in the present study. These hypotheses could be tested by comparing learning-related activations during immediate versus later (few days or months later) retrieval sessions in the same group of children, or comparing adults and children populations on this specific task. Higher levels of task difficulty may also explain the increased learning-related activity found over bilateral orbito-frontal regions, in addition to the right temporal regions. Indeed, highly challenging conditions for semantic processing may increase functional connectivity between ventral inferior frontal and middle temporal gyri [62]. Increased activation in inferior and middle-orbital frontal gyri was also found in elderly participants required to name high vs. low familiarity objects, especially in the low familiarity (i.e. higher difficulty) condition [63]. Likewise, Blumenfield et al. (2006) disclosed higher activations in low than high accuracy performers in ventral inferior frontal areas during visual and auditory semantic association tasks in which 9–12 years old children had to judge whether a third stimulus was semantically related to the previous one [64]. Further studies should attempt at disentangling these potential contributions to the observed effects.

Finally, we have found functional learning-related modulations of the ERFs over the right parietal lobule in a 530–690 msec time window. Accordingly, two picture-naming MEG studies [10], [11] have associated parietal magnetic responses with the lexical retrieval of newly learned words associated to real unknown objects. Parietal learning-related activations were interpreted as being due to the higher phonological encoding and motor preparation processes needed for the production of a newly learned name, as compared to the generation of the relatively stereotyped response (e.g. ‘‘object”) provided for an unknown object. Also in our study participants had to provide a stereotyped response (i.e. “I skip”) when confronted with unknown objects to-be-learned at session 1, whereas the response was more complex at session 2 for the same objects now associated with a magic definition of the object's function. Consequently, the different types of delayed oral responses required between sessions for LNO stimuli might have involved working memory subsystems at different levels, resulting in enhanced activation in the parietal region for newly learned non-objects. Although phonological access and motor preparation for articulation processes are most commonly linked with activity in the inferior frontal cortex [65]–[68], more than in parietal areas, and our design was made in such a way that oral responses were provided only after the recording period of interest to avoid contamination, these elements should be considered in the interpretation of the observed changes in learning-related ERFs in parietal areas. Alternatively, parietal activity was also linked with objects manipulation, although mostly in the left hemisphere [7], [15], [17]. From this perspective, learning functions for a novel object may have created an associated sensori-motor representation, eventually leading to the recruitment of parietal regions during the retrieval of the object's magic functions. To sum up, we have found that learning novel functions for unknown objects is associated with increased long-lasting and late neuromagnetic components, extending 500 to 800 msec peristimulus time initially in medial orbitofrontal and right parietal then in right medial, middle and inferior temporal regions. Especially, we propose that increased evoked magnetic fields over the right temporal lobe may support the challenging task of rapidly creating new semantic representations about the functionalities of novel, unknown objects. Future studies should determine whether such patterns are specific to the creation of functional attributes in semantic representations.

## Supporting Information

Supporting Information S1 **Supplemental details on the methods as well as supplemental Tables and Figures.** Figure S1, Learning-related changes in evoked-related fields over the left frontal region (analysis in sensor space). Grand average time courses of ERFs for LNO and UNO non-objects at S1 (LNO: black hyphenated line, UNO: gray hyphenated line) and S2 (LNO: black line; UNO: gray line). Significant differences over these sensors are identified 552–656 msec post-stimulus onset. Figure S2, Learning-related changes in evoked-related fields over (a) the right frontal and (b) right anterior temporal regions (analysis in sensor space). Grand average time courses of activation for the LNO and UNO stimuli at S1 (LNO: black hyphenated line, UNO: gray hyphenated line) and S2 (LNO: black line; UNO: gray line). Significant differences over these sensors are identified around 724 and 572 msec post-stimulus onset, respectively, but data inspection did not consistently reveal obvious differences in the time course and amplitude of evoked magnetic responses for LNO as compared to never taught non objects (UNO). Table S1, Random analysis results in the source space. Brain regions showing higher activity in the pre-learning session compared to the post-learning session during the MEG task (LNO S1>S2 masked exclusively for between-sessions repetition effects for untaught non objects (exclusive mask UNO S2 vs. S1)). Table S2, Common patterns of activation to both S1 and S2. Null conjunction analysis revealing patterns of activation common to both S1 and S2 in the source space during object identification. Table S3, Random analysis results in the source space. Main effect of brain activity during object identification in each session. LNO S1; S2.
(DOC)
