# Supplementary material for: MEG Correlates of Learning Novel Objects Properties in Children
Source: PLoS One. 2013 Jul 31;8(7):e69696. doi: 10.1371/journal.pone.0069696 (PMC3729701; doi:10.1371/journal.pone.0069696)
Supplement: Supporting Information S1 — Supplemental details on the methods as well as supplemental Tables and Figures. Figure S1, Learning-related changes in evoked-related fields over the left frontal region (analysis in sensor space). Grand average time courses of ERFs for LNO and UNO non-objects at S1 (LNO: black hyphenated line, UNO: gray hyphenated line) and S2 (LNO: black line; UNO: gray line). Significant differences over these sensors are identified 552–656 msec post-stimulus onset. Figure S2, Learning-related changes in evoked-related fields over (a) the right frontal and (b) right anterior temporal regions (analysis in sensor space). Grand average time courses of activation for the LNO and UNO stimuli at S1 (LNO: black hyphenated line, UNO: gray hyphenated line) and S2 (LNO: black line; UNO: gray line). Significant differences over these sensors are identified around 724 and 572 msec post-stimulus onset, respectively, but data inspection did not consistently reveal obvious differences in the time course and amplitude of evoked magnetic responses for LNO as compared to never taught non objects (UNO). Table S1, Random analysis results in the source space. Brain regions showing higher activity in the pre-learning session compared to the post-learning session during the MEG task (LNO S1>S2 masked exclusively for between-sessions repetition effects for untaught non objects (exclusive mask UNO S2 vs. S1)). Table S2, Common patterns of activation to both S1 and S2. Null conjunction analysis revealing patterns of activation common to both S1 and S2 in the source space during object identification. Table S3, Random analysis results in the source space. Main effect of brain activity during object identification in each session. LNO S1; S2. (DOC) [file pone.0069696.s001.doc]

Supporting Information S1 **to “MEG Correlates of Learning Novel Objects Properties in Children.”**

Charline Urbain^1,2^, Mathieu Bourguignon^2^, Marc Op de Beeck^2^, Rémy Schmitz^1^, Sophie Galer^1,2^, Vincent Wens^2^, Brice Marty^2^, Xavier De Tiège^2^, Patrick Van Bogaert^2^, and Philippe Peigneux^1^

^1^ UR2NF - Neuropsychology and Functional Neuroimaging Research Group at CRCN - Center for Research in Cognition and Neurosciences and UNI - ULB Neurosciences Institute, Université Libre de Bruxelles (ULB), Brussels, Belgium.

^2^ LCFC - Laboratoire de Cartographie Fonctionnelle du Cerveau, Hôpital Erasme, Brussels, Belgium.

**(SI) Methods**

*Picture-definition task (MEG sessions S1 and S2)*

The main goal of the present study was to characterize at the temporal and spatial levels the neurophysiological patterns supporting in children the learning of functional properties for novel objects (Fig. 1A). To do so, we measured evoked MEG responses subtending visual processing of novel non-objects during a picture-defining task occurring immediately before [session 1, (S1)] and after [session 2, (S2)] a behavioural verbal learning session. Given the somewhat boring and un-ecological character of the pre-learning session (in which by definition children were not supposed to know the novel objects presented), we choose to add a condition during which familiar objects (FO) were presented, to make the task more enjoyable and provide children an opportunity to engage in semantic retrieval already at S1. Instructions were as follows: *“If you know the object’s utility, you must say aloud its name or provide a brief definition of its function so I can be sure that you know it. If you do not know at all this object, simply say “I skip” and I will show you the next object*. In S1, children had to name and/or briefly define 150 objects (50 familiar objects [FO] and 100 unknown non objects (50 non objects to be learned [LNO] after S1, and 50 unknown non objects [UNO] not assigned to the learning session; Fig.1A). Children had to provide an answer for each item.

During each MEG session (S1 and S2), all stimuli were presented twice to enhance signal-to-noise ratio, in a randomized order. During MEG recording, stimuli appeared on a back-projection screen located 1 m in front of the child face, fitting in a 3° x 3° area in the central visual field. Each picture was shown for 150 msec, followed by a 850 msec blank screen then for 1000 msec by a question mark prompting the child to say aloud its response (name/or definition of the object, or "skip"). The next trial was initiated 1000 msec after the subject's response (Fig.1B). At any time during the experiment, children were allowed to ask for a break.

After S1, a computerized learning phase occurred outside of MEG environment (see below). After the end of the behavioural learning phase, all participants were retested at S2 while performing again the overt picture naming/defining task in the MEG environment. S2 occurred in exactly the same way than S1 except that children were now able to define at least 75% of the newly learned non objects [LNO].

*Learning Phase*

After MEG S1, children had to learn semantic information (short “magical” definitions) associated to 50 LNO non objects, randomly selected from the 100 unfamiliar non objects previously presented in S1. Participants were trained until a specific criterion was reached (75% of the definitions mastered) to ensure successful retrieval of a sufficient number of LNO stimuli to analyse evoked MEG responses associated to the learning of novel object’s functions. The learning session occurred outside of the MEG environment and was made as ecological as possible. The experimenter told to the child that she/he was participating into a game. In this game she/he was a secret agent and needed to learn the functionalities of magic objects that would be useful for further missions. The learning session occurred in two steps: the learning phase in itself, during which the experimenter taught the 50 definitions associated to 50 LNO stimuli, 5 by 5, across 10 learning blocks. Then, a final retrieval testing occurred during which all stimuli were presented and the experimenter assessed the knowledge gained by the children.

For each block of 5 stimuli in the learning phase, the experimenter said aloud the definition associated to each LNO object, which was shown on the computer screen with no explicit time limit. To enhance the ecologic character of the learning task, a name (non-word) was provided during the presentation of each LNO object during the learning session, although children were only explicitly prompted to learn the definition (e.g. “*This object is a “kuvlɔ̃”, you can use it to see through the walls”*). Each presentation was followed by a cued recall testing (identical to the picture naming/defining task in the MEG session; Fig. 1B), during which the child was asked to recall orally at least the definition of the object. If the answer was correct, the next object appeared. If the answer was incorrect, a feedback was provided with the correct definition. When all 5 objects and their name and definition had been presented, an immediate retrieval was proposed to ensure that the child had learnt at least for the short term the material within the block. Feedback was provided again in case of incorrect answer. This procedure was repeated for each of the 10 blocks.

When presentation of all LNO stimuli was completed, a final retrieval was proposed to ensure that the child had learnt at least 75% of the material. He was again asked to recall the objects definitions using the same cued recall procedure as during learning and the MEG sessions. Feedback was not given at this stage in case of incorrect answer. If the 75% criterion was not reached, the unlearned objects were presented again in a second learning phase after which all the objects were retested until at least 75% of the objects were successfully learnt. FO and UNO items were not presented during this behavioural learning session, keeping total learning time in acceptable conditions (i.e. less than 1 hour).

**Figure S1**


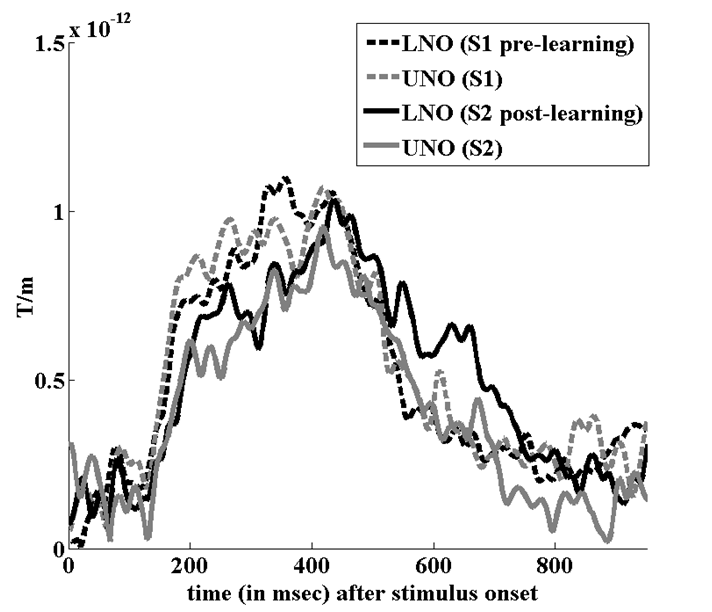


**Figure S1. Learning-related changes in evoked-related fields over the left frontal region (analysis in sensor space).** Grand average time courses of ERFs for LNO and UNO non-objects at S1 (LNO: black hyphenated line, UNO: gray hyphenated line) and S2 (LNO: black line; UNO: gray line). Significant differences over these sensors are identified 552-656 msec post-stimulus onset.

**Figure S2**

a. b.

**
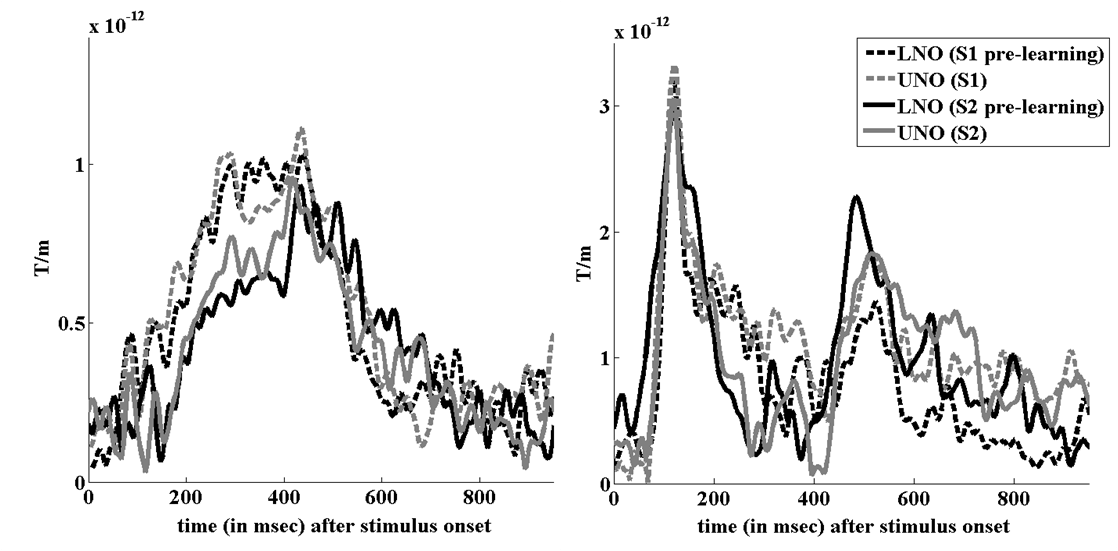
**

**Figure S2. Learning-related changes in evoked-related fields over (a) the right frontal and (b) right anterior temporal regions (analysis in sensor space).** Grand average time courses of activation for the LNO and UNO stimuli at S1 (LNO: black hyphenated line, UNO: gray hyphenated line) and S2 (LNO: black line; UNO: gray line). Significant differences over these sensors are identified around 724 and 572 msec post-stimulus onset, respectively, but data inspection did not consistently reveal obvious differences in the time course and amplitude of evoked magnetic responses for LNO as compared to never taught non objects (UNO)**.**

**Table S1**

Table S1. Random analysis results in the source space. Brain regions showing higher activity in the pre-learning session compared to the post-learning session during the MEG task (LNO S1>S2 masked exclusively for between-sessions repetition effects for untaught non objects (exclusive mask UNO S2 vs. S1)).

| Time window (ms) | *Voxel-level* |  |  |  |  |  | *Cluster extent* |  |
| --- | --- | --- | --- | --- | --- | --- | --- | --- |
|  | Region | Side | x | *y* | *z* | *Z* | ^K^E *cluster* | *Additional activated regions in the cluster* |
| 530-690 | Posterior occipito-temporal | Left | -40 | -68 | -24 | 3.21 | 1441 | Bilateral fusiform gyrus |
|  | Posterior occipito-temporal | Right | 48 | -56 | -6 | 2.79 | 591 |  |
| 700-760 | Posterior parieto-occipital | Right | 30 | -58 | 26 | 3.36 | 828 |  |

Brain regions showing higher activity in the pre-learning session compared to the post-learning session during the MEG task (LNO S1>S2) at the population level (all participants) in the source space. Time windows of sources reconstruction corresponded to the temporal epochs (± 20msec) during which significant learning-related activations (LNO S1<S2, see table 1) were statistically identified in the sensor space. x, y, and z are standard MNI coordinates (mm). Z = Z-statistic value. KE cluster = cluster extent of the activation (in number of voxels). All reported activations are statistically significant at the voxel-level, p^uncorr^≤.003.

**Table S2**

Table S2. Null conjunction analysis revealing patterns of activation common to both S1 and S2 in the source space during object identification.

| Time window (ms) | *Voxel-level* |  |  |  |  | *Cluster extent* |  |
| --- | --- | --- | --- | --- | --- | --- | --- |
|  | Region | Side | x | *y* | *z* | *Z* | ^K^E *cluster* |
| 530-690 | Inferior anterior temporal gyrus | Left | -40 | -2 | -30 | 4.51 | 19 |
|  | Inferior anterior temporal gyrus | Right | 42 | -4 | -30 | 4.37 | 10 |
|  | Inferior medial parietal gyrus | Left | -22 | -30 | 38 | 4.46 | 7 |
|  | Inferior medial parietal gyrus | Right | 26 | -22 | 36 | 4.47 | 7 |
| 700-760 | Medio-temporal gyrus | Right | 42 | -26 | -8 | 4.72 | 22 |
|  | Inferior temporal gyrus | Left | -40 | -8 | -24 | 4.42 | 60 |
|  | Inferior temporal gyrus | Right | 56 | -4 | -34 | 4.55 | 66 |

Null conjunction analysis revealing patterns of activation in source space common to both S1 and S2 during object identification. x, y, and z are standard stereotactic coordinates (mm). Z = Z-statistic value. Time windows of sources reconstruction corresponded to the temporal epochs (± 20msec) during which learning-related activations (LNO S1<S2, see table 1) are statistically identified in the sensor space. All activations are statistically significant at the voxel level at *p* < .05 (corrected for multiple comparisons in the whole brain volume).

**Table S3**

Table S3. Random analysis results in the source space. Main effect of brain activity during object identification in each session. LNO S1; S2.

| Time window (ms) | | *Voxel-level* |  |  |  |  | *Cluster extent* |  |
| --- | --- | --- | --- | --- | --- | --- | --- | --- |
|  | | Region | Side | x | *y* | *z* | *Z* | ^K^E *cluster* |
| 700-760 | S1 | Middle and inferior temporal gyrus | Left | -42 | -10 | -24 | 5.10 | 1230 |
|  |  |  | Right | 42 | -26 | -8 | 4.72 | 22 |
|  | S2 | Middle and inferior temporal gyrus | Left | -40 | -8 | -24 | 4.42 | 64 |
|  |  |  | Right | 42 | -26 | -8 | 5.88 | 2863 |

Peak voxel in the temporal region for each session associated to the presentation of LNO objects during the MEG task (LNO S1; S2) at the population level (all participants) in the source space. Time windows of sources reconstruction corresponded to the temporal epochs (± 20msec) during which significant learning-related activations (LNO S1<S2) were statistically identified in the sensor space. x, y, and z are standard MNI coordinates (mm). Z = Z-statistic value. KE cluster = cluster extent of the activation (in number of voxels). All reported activations are statistically significant at the voxel-level, p^corr^ <.05 for multiple comparisons in the whole brain volume.
